# Oncology biosimilars: New developments and future directions

**DOI:** 10.1002/cnr2.1720

**Published:** 2022-10-04

**Authors:** Rinda Devi Bachu, Mariam Abou‐Dahech, Swapnaa Balaji, Sai H. S. Boddu, Samson Amos, Vishal Singh, R. Jayachandra Babu, Amit K. Tiwari

**Affiliations:** ^1^ Department of Pharmacology and Experimental Therapeutics College of Pharmacy & Pharmaceutical Sciences, University of Toledo Toledo Ohio USA; ^2^ College of Pharmacy and Health Sciences Ajman University Ajman UAE; ^3^ Center of Medical and Bio‐allied Health Sciences Research Ajman University Ajman UAE; ^4^ Department of Pharmaceutical Sciences Cedarville University School of Pharmacy Cedarville Ohio USA; ^5^ Department of Nutrition Pennsylvania State University State College Pennsylvania USA; ^6^ Department of Drug Discovery & Development Harrison School of Pharmacy, Auburn University Auburn Alabama USA; ^7^ Department of Cell and Cancer Biology College of Medicine & Life Sciences, University of Toledo Toledo Ohio USA

**Keywords:** bevacizumab, biosimilars, epoetins, filgrastim, new developments, oncology, pegfilgrastim, rituximab

## Abstract

Biologicals have become an integral part of cancer treatment both as therapeutic agents and as supportive care agents. It is important to know that biologics are large, complex molecular entities requiring extensive immunogenicity testing and pharmacovigilance strategies to ensure no immune response is evoked in the body. Oncology's pharmacological market is dominated by biologics; however, their high development and manufacturing costs are burdensome to health care systems. Biologics being the most expensive prescription drugs on the market limit the accessibility for necessary treatment in the case of many patients. As biologics patents expire, the development of biosimilars is underway in an effort to lower costs and enable patients to access new cancer therapies. Regulatory guidelines for biosimilars have now been established and are constantly being revised to address any issues, facilitating their robust development. Moreover, many scientific societies offer guidance to help stakeholders better understand current regulations and biosimilar's safety. Despite the potential cost benefits, lack of knowledge about biosimilars, and the possibility of immunogenicity have created an uncertain environment for healthcare professionals and patients. In this review, we provide an overview of relevant legislation and regulations, pharmacoeconomics, and stakeholder perceptions regarding biosimilars. The article also describes biosimilars in development, as well as the ones currently available on the market.

## 1 INTRODUCTION

Biologics are generally large complex molecules produced through biotechnology in a living system such as microorganism, plant cell, or animal cell. These products are used to diagnose, prevent, treat, and cure medical conditions.^1^ Biosimilars are biological drugs that are designed to be highly similar to the existing marketed biologics.^2^ The high level of similarity to the originator biologic is defined in terms of physicochemical characteristics, efficacy, and safety as outlined by the respective regulatory authorities.^3^, ^4^ A generic drug is a medication that has the same active ingredients and provides the same clinical benefits as that of a brand name drug. It is created to have an identical dosage form, safety, strength, route of administration, quality, performance characteristics, and intended use.^1^ However, biosimilars are not a generic version of biologics, as it is not possible to develop an identical biochemical entity. This is mainly due to the inherent complexity of the proteins and their associated manufacturing processes.^5^ Inherent variation is common within each lot and between lots during the manufacturing of biologics as well as biosimilars. Both Biosimilars and generics are approved through different abbreviated pathways that do not require extensive clinical studies.^6^ So far, 29 biosimilars for various indications have been approved in the United States^7^ whereas 64 biosimilars were approved in Europe.^8^ The European Medical Agency (EMA) was the first to approve a biosimilar in 2006 and to provide guidance for biosimilar development and approval.^9^ However, the pathway for marketing biosimilars in the United States has had several barriers.^10^ Even though the patent protection of several originator biologics was close to the expiration date, the market competition that was seen with chemical drugs through generics did not occur with biosimilars.^11^ Generics have been able to be marketed in the United States since 1984 due to the established abbreviated pathway through the Hatch‐Waxmann Act.^12^ However, with biologics the FDA lacked a clear regulatory pathway for the approval of biosimilars until 2010. This was one of the main reasons for the slow adoption of biosimilars in the US when compared to Europe.^13^ Moreover, the marketing launch of biosimilars in the US is delayed by patent infringement lawsuits, exclusionary contracts, and anticompetitive tactics of brand name manufacturers.^14^

## 2 THE NEED FOR BIOSIMILARS IN CANCER

Cancer is among the leading causes of death worldwide. Globally, cancer accounts for about one in every six deaths, which is more than HIV, tuberculosis, and malaria combined.^15^ In 2020, there were about 19.3 million estimated new cases and 10 million cancer‐related deaths worldwide. Among these deaths, one‐quarter of the cases occur in low‐ and medium‐Human Development Index countries, which lack resources and medical systems to address the disease burden.^16^ By 2040, the global cancer burden is expected to increase to an estimated 27.5 million cases and 16.3 million deaths based on the aging and growth of the population.^17^ The National Cancer Institute estimates the direct medical costs related to cancer treatment in the United States were $183 billion in 2015 and are expected to increase to $246 billion by 2030, a 34% raise.^18^ However, owing to the advances in personalized treatments and inflation, this increase is likely to be an underestimation.^19^, ^20^ With the advent of biosimilars, market competition is on the rise which can help in increasing the accessibility and decreasing the cost burden to cancer patients.

### 2.1 Stake holders' perceptions on biosimilars/barriers to implementation

There are numerous obstacles to the integration of biosimilars into oncology treatment. One major barrier is the patient and prescriber perception of biosimilars. Survey responses collected from 1201 US physicians across specialties by the biosimilars Forum through SERMO (global social media/network organization for physicians) indicated knowledge gaps among physicians. Lack of awareness about biologics, biosimilars, the approval process for biosimilars, safety & immunogenicity, interchangeability, and substitution of biosimilars was observed.^21^ Another survey involving 500 US‐based hematologists and oncologists also indicated critical education gaps. Almost 49% of the respondents were not familiar with the concept of extrapolation and 81% of respondents were hesitant to prescribe biosimilars until an average sales price (ASP) was established. However, 77% of respondents were receptive to receiving communications about biosimilars from professional organizations like ASCO.^22^ Moreover, there is a growing concern that regulatory guidelines of generics may be applied to biologics, which has led several states to amend older laws to address the complex molecular characteristics of biologic products and biosimilars.^23^

Given the novelty of biosimilars and their reduced emphasis on clinical testing, there is a great need for education among prescribers and patients.^24^ American Society of Clinical Oncology (ASCO) provides information and guidance to the oncology community on the use of biosimilars, their safety & efficacy, interchangeability, substitution, regulatory considerations, and prescriber & patient education. CancerLinQ, an integrated real‐time data resource also provides valuable information on the use of biosimilars and their effectiveness.^23^ The FDA also offers educational webinars and presentations to help clinicians better understand current regulations and biosimilar's safety.^25^ A few other scientific societies including National Comprehensive Cancer Network (NCCN),^26^ European Society for Medical Oncology (ESMO)^27^ also provide guidance on biosimilars. Additionally, European Public Assessment Reports (EPARs) published by the EMA help clinicians in evaluating the appropriate use of biosimilars in Europe.^28^ In the case of patients, the primary education source is the treating physician.^29^ Several patient advocacy groups including CancerCare,^30^ Susan G. Komen,^31^ Global Colon Cancer Association,^32^ and so forth, also provide a broad range of educational materials tailored for patient use to facilitate their understanding and acceptance of biosimilars.

#### 2.1.1 Overview of biosimilar legislation and regulation

Historically in the United States, biologics were regulated by the Public Health Hygienic Laboratory, a precursor of NIH, which was then transferred to the Bureau of Biologics at the FDA in 1972.^33^ After a decade later, the Bureau of Drugs and Bureau of Biologics were merged into a single entity to form National Center for Drugs and Biologics (NCDB).^34^ However, in 1987 the Center for Drugs and Biologics was divided back into the Center for Drug Evaluation and Research (CDER) and the Center for Biologics Evaluation and Research (CBER).^35^ The jurisdictional responsibilities of the two centers were assigned through the Intercenter Agreement issued by the FDA in 1991.^36^ Traditional biologics including vaccines, blood, blood products, allergenic extracts, certain devices, and test kits are regulated by CBER. The center also regulates gene & cellular therapy products and tissue transplants from human and non‐human sources.^37^ CDER on the other hand regulates prescription, over‐the‐counter & generic drugs,^38^ and most therapeutic biologics including monoclonal antibodies, cytokines, growth factors, enzymes, immunomodulators, and so forth.^39^

Most of the biological products were approved under the Public Health Service Act (PHSA) while some of them are licensed as drugs under the Federal Food, Drug, and Cosmetic Act (FFDCA).^40^ In 2010, Congress established an abbreviated licensure pathway for biological products that demonstrated to be biosimilar or interchangeable to a previously licensed biological product. This new regulatory authority for FDA was accomplished through the Biologics Price Competition and Innovation Act (BPCIA) of 2009, which was enacted as Title VII of the Patient Protection and Affordable Care Act (ACA).^11^ As a part of the implementation of BPCIA, three draft guidances on the development of biosimilars were released by the FDA in 2012^41^ and the final versions were released in 2015.^42^ The BPCIA has also set periods of regulatory exclusivity for brand name biologics and biosimilars as well as laid procedures for resolving patent disputes.^11^ Biologics are offered 12 years of exclusivity during which the FDA cannot approve any biosimilar or interchangeable product referencing the brand name biologic. However, a BLA (Biologics License Application) of a biosimilar or interchangeable product can be submitted after 4 years from the date on which the reference product was first licensed.^43^ A BLA can be submitted directly by an applicant or through a legal entity involved in the manufacturing, who is responsible for product compliance according to the established standards. Form 356 h is to be submitted along with the BLA, which includes information about the applicant, product, manufacturing process, preclinical & clinical studies, and draft labeling of the product.^44^ Also, effective from March 23, 2020, biological products under BPCIA, which were previously approved as drugs under 505 of the FFDCA are transitioned to biological licenses under section 351 of the PHSA.^45^

Over the years, the FDA has released additional guidance on a variety of other areas related to biosimilars and all of these documents can be accessed through the FDA website.^42^ The agency's database “Purple Book” contains information about all FDA‐licensed biologics regulated by the CDER including their biosimilars and interchangeable products. In‐depth information about the date on which the biological product was licensed, if the biological product has proven to be a biosimilar or interchangeable to an already licensed biological drug, and the expiration dates of applicable exclusivities of the reference biologics can be obtained. Also, the database provides information about licensed products regulated by the CBER.^46^

On the other hand, guidelines for the regulation of medicines in the European Union (EU) are very well established. A dedicated pathway for the development and approval of biosimilars was introduced in 2004.^47^ General guidelines on biosimilars were issued by EMA to introduce the concept and to provide biosimilar manufacturers with a user guide containing relevant scientific information.^48^ In the EU, biologics are offered 8 years of exclusivity during which a biosimilar referencing the brand name biologic cannot be marketed.^4^ Biotechnology products including biosimilars are approved by the EMA through a marketing authorization application (MAA) following a centralized procedure.^47^ This procedure authorizes the manufacturers to market their products throughout the European Economic Area (EEA) with a single marketing authorization application. EEA includes all EU member states, and three countries of the European Free Trade Association (EFTA)‐Iceland, Liechtenstein, and Norway.^49^ The MAA's for biosimilars are evaluated by EMA's scientific committees including Committee for Medical Products for Human Use (CHMP), Pharmacovigilance Risk Assessment Committee (PRAC) as well as EU experts & specialists on biological medicines (Biologics Working Party) and biosimilars (Biosimilar Working Party).^47^ The scientific opinion obtained after the EMA's evaluation is recommended to the European Commission, which ultimately decides if an EU‐wide marketing authorization must be granted. Once approved, the decision of the commission is published in the Community Register of medicinal products for human use. In addition, the EMA also publishes a European public assessment report (EPAR) for each application that has been granted/refused a marketing authorization.^49^ The complete list of centrally authorized biosimilars approved to date can be accessed from the EMA's website.^8^

#### 2.1.2 Biosimilars in oncology

Currently, there are only a few biosimilars approved for cancer treatment and supportive care. Biosimilars are available for monoclonal antibodies (mAb) including Rituximab, Trastuzumab & Bevacizumab, and supportive agents including filgrastim, pegfilgrastim, epoetin α & epoetin ζ.^50^

##### Filgrastim and pegfilgrastim

**FIGURE 1:**
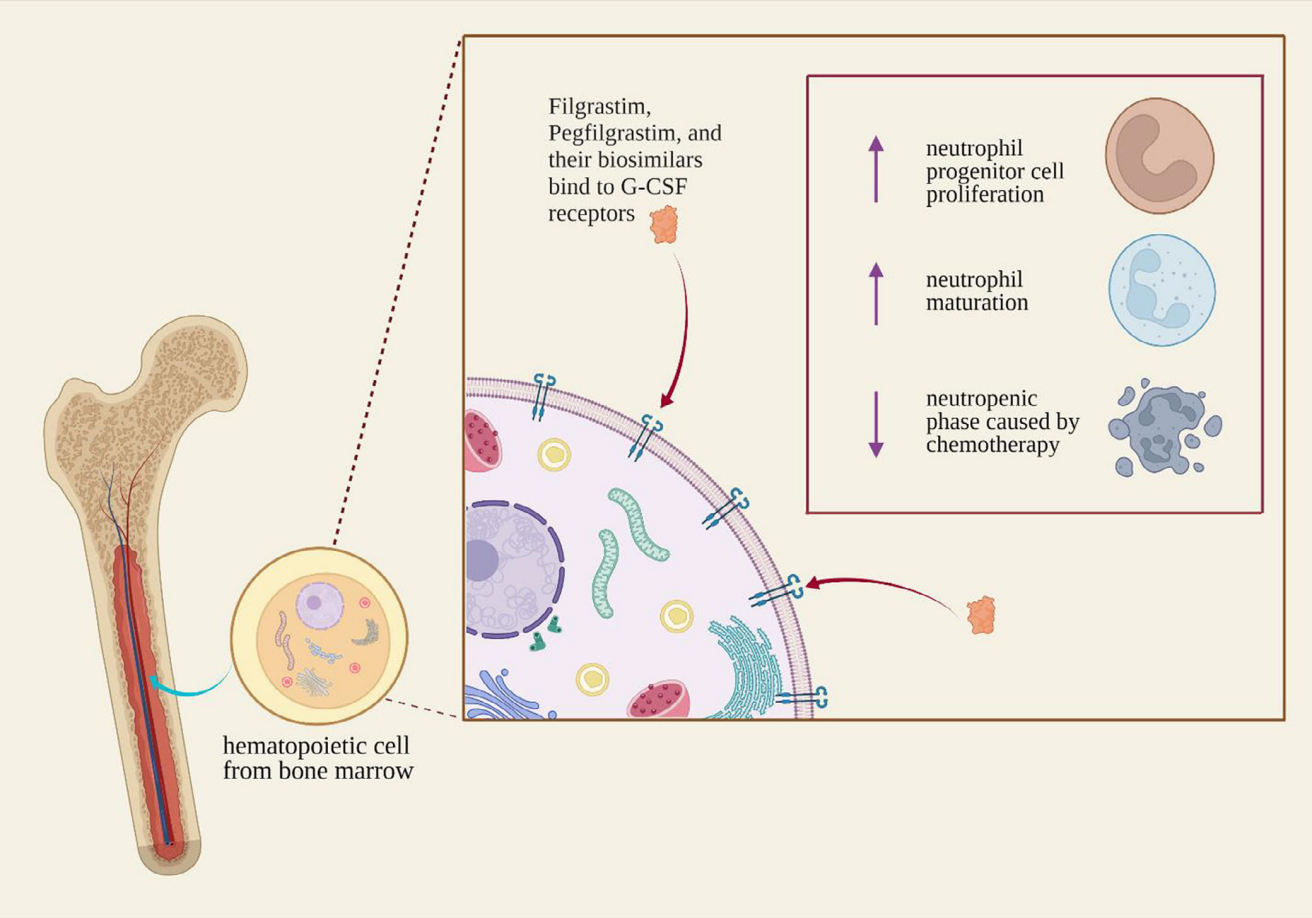
A visual representation of the effects filgrastim, pegfilgrastim, and their biosimilars have upon binding to granulocyte colony stimulating factor receptors.

**FIGURE 2:**
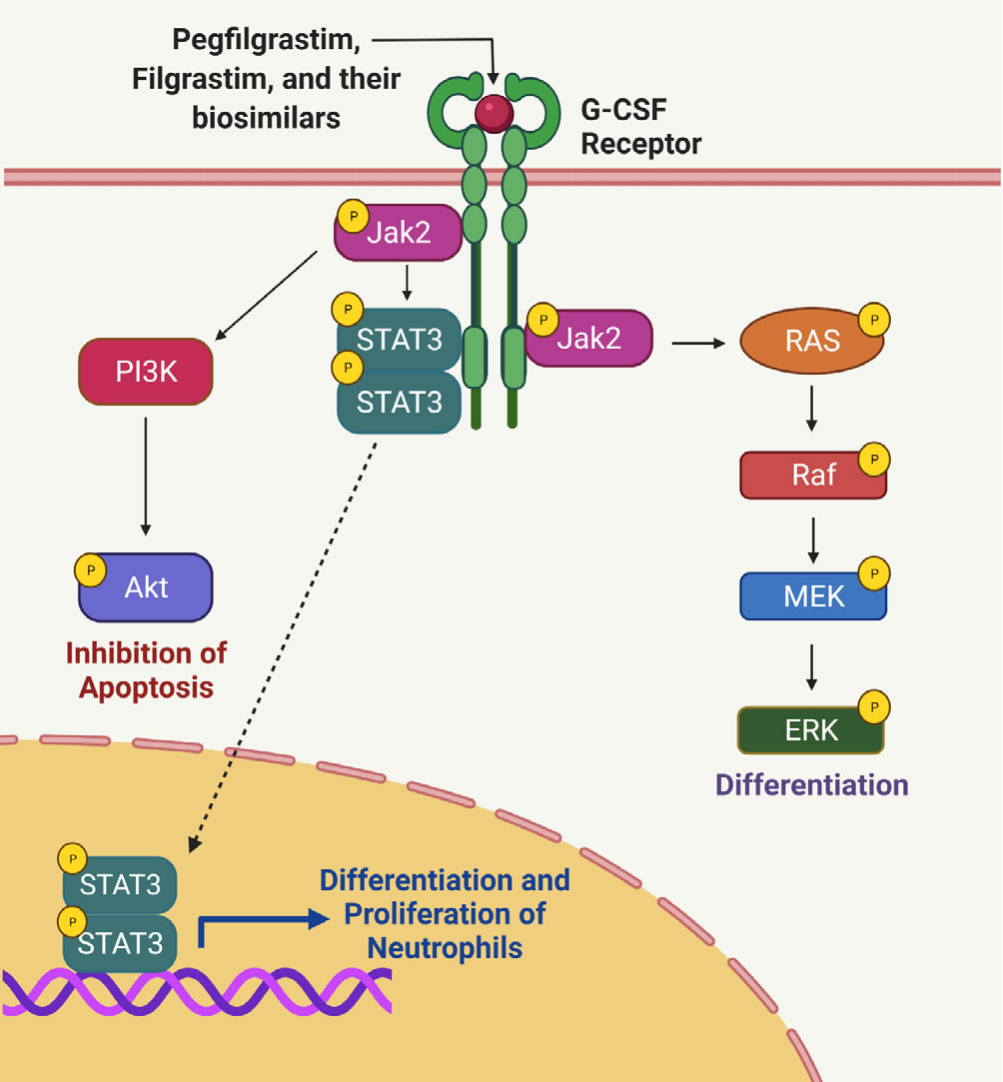
Pegfilgrastim, filgrastim and their biosimilars' mechanism of action. Once bound to the granulocyte colony stimulating factor receptor, the JAK–STAT signaling pathway is activated, leading to neutrophil survival, proliferation, and differentiation.

The first‐ever biosimilar product to be marketed in the United States was Zarxio® (filgrastim‐sndz) and was approved by FDA Y.T.T March 2015.^51^ Later in 2018, Nivestym® (filgrastim‐aafi) was approved, both of these biosimilars can be used for the same indications as the reference drug, Neupogen® (Filgrastim).^52^ Filgrastim is a recombinant granulocyte colony‐stimulating factor (G‐CSF) that regulates neutrophil production from bone marrow. Filgrastim is used to reduce febrile neutropenia in patients with non‐myeloid malignancies receiving myelosuppressive anticancer agents or myeloablative chemotherapy followed by bone marrow transplantation. It is also used in patients with acute myeloid leukemia receiving induction or consolidation chemotherapy for reducing the time of neutrophil recovery and the duration of fever.^53^ In Europe, nine biosimilars of Filgrastim are approved by the EMA including Accofil®,^54^ Biograstim®^55^ Filgrastim Hexal®,^56^ Filgrastim Ratiopharm®,^57^ Grastofil®,^58^ Nivestim®,^59^ Ratiograstim®,^60^ Tevegrastim®^61^ and Zarzio®.^62^ However, the marketing of Biograstim® and Filgrastim Ratiopharm® was withdrawn by the EMA at the request of their respective marketing authorization holders.^55^, ^57^ Two other G‐CSFs that are commonly used for treating chemotherapy‐induced neutropenia (CIN) include pegfilgrastim and lenograstim. Filgrastim and lenograstim are short‐acting G‐CSFs that are injected daily during chemotherapy while pegfilgrastim is a long‐acting G‐CSF, administered once per chemotherapy cycle.^63^ Pegfilgrastim has an additional polyethylene glycol unit, which causes an increase in the size of the molecule, thereby prolonging the half‐life of the drug.^64^ Once bound to G‐CSF receptors, filgrastim, pegfilgrastim, lenograstim, and all biosimilars act to increase the proliferation and maturation of neutrophils thereby decreasing the risk for neutropenia as seen in Figure 1. The JAK–STAT signaling pathway is activated and results in the translocation of JAK3 to the nucleus. Once in the nucleus, JAK3 binds to DNA and activates transcription linked to neutrophil proliferation^65^ as seen in Figure 2. Eight approved biosimilars for pegfilgrastim are available in Europe including Pelgraz®,^66^ Udenyca®,^67^ Fulphila®,^68^ Pelmeg®^69^ Ziextenzo®^70^ Grasustek®^71^ Cegfila®,^72^ and Nyvepria®.^73^ Whereas in United States, for Neulasta® (pegfilgrastim) four biosimilars are approved: Fulphila® (pegfilgrastim‐jmdb)^74^ Udenyca® (pegfilgrastim‐cbqv),^75^ Ziextenzo® (pegfilgrastim‐bmez)^76^ and Nyvepria® (pegfilgrastim‐apgf).^77^ To, date no biosimilars for lenograstim are available.

##### Epoetins

**FIGURE 3:**
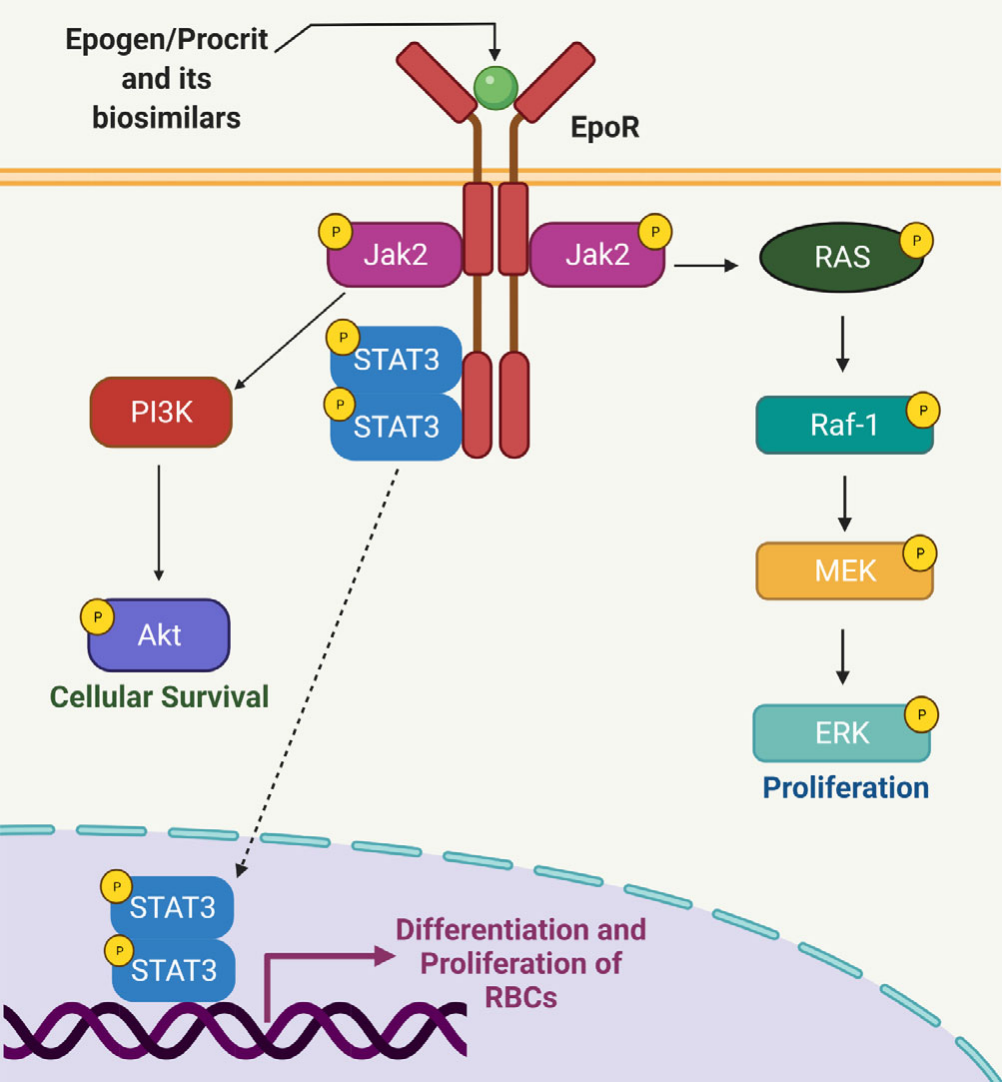
Epogen/Procrit and its biosimilars' mechanism of action. Once bound to the erythropoietin receptor, the JAK–STAT signaling pathway is activated, leading to red blood cell survival, proliferation, and differentiation.

**FIGURE 4:**
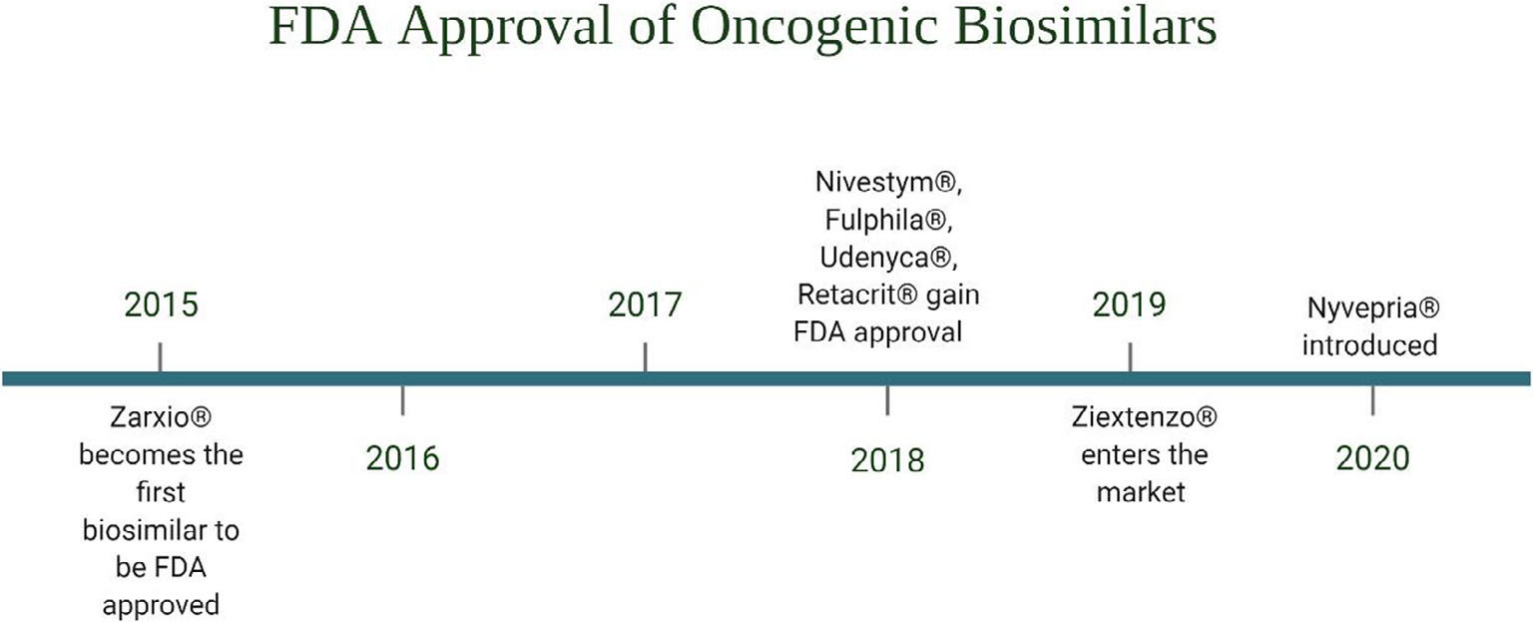
Timeline: A timeline about FDA approval of biosimilars of oncogenic biologics.

Epoetins are used for treating chemotherapy‐induced anemia (CIA), reducing the need for blood transfusions thereby improving the quality of life. These are similar to erythropoietin hormone, secreted by the kidneys that stimulate red blood cell production (erythropoiesis) in the bone marrow and are also referred to as erythropoiesis‐stimulating agents (ESAs).^78^, ^79^ Epoteins and their biosimilars bind to the erythropoietin receptor and activate the JAK–STAT signaling pathway. JAK3 translocates to the nucleus and binds to DNA activating transcription linked to red blood cell proliferation^80^ (Figure 3). Five epoetin biosimilars are approved in Europe including three epoetin α (EPO‐ α) biosimilars: Abseamed®,^81^ Binocrit®,^82^ Epoetin‐ α hexal®,^83^ and two epoetin ζ (EPO‐ζ) biosimilars Retacrit®^84^ & Silapo®.^85^ In the United States only one ESA agent, Retacrit® (epoetin alfa‐epbx)^86^ has been approved for the reference drug, Epogen®/Procrit® (EPO‐α). Both EPO‐α and EPO‐ζ have been approved for treating chemotherapy‐induced and symptomatic anemia in patients with solid tumors, malignant lymphoma, or multiple myeloma.^87^, ^88^ Figure 4 represents the timeline of FDA approval of supportive cancer care biosimilars. Tables 1 and 2 lists the biosimilar drugs approved for supportive cancer care by the FDA and EMA respectively.

**TABLE 1:** FDA approved supportive care biosimilars in oncology

Reference Biologic (Active substance)	Reference Biologic manufacturer(s)	Biosimilar (active substance)	Biosimilar manufacturer	Approval date
Neupogen® (Filgrastim)	Amgen Inc	Zarxio® (filgrastim‐sndz)	Sandoz Inc	2015
		Nivestym® (filgrastim‐aafi)	Hospira Inc	2018
Neulasta® (pegfilgrastim)	Amgen Inc	Fulphila® (pegfilgrastim‐jmdb)	Mylan GmbH	2018
		Udenyca® (pegfilgrastim‐cbqv)	Coherus BioSciences, Inc	2018
		Ziextenzo® (pegfilgrastim‐bmez)	Sandoz Inc	2019
		Nyvepria® (pegfilgrastim‐apgf)	Hospira Inc	2020
Epogen/Procrit (epoetin α)	Amgen Inc/Janssen Biotech Inc	Retacrit® (epoetin alfa‐epbx)	Hospira Inc	2018

**TABLE 2:** EMA approved supportive care biosimilars in oncology

Reference Biologic (Active substance)	Reference Biologic manufacturer(s)	Biosimilar (active substance)	Biosimilar manufacturer	Approval date
Neupogen® (Filgrastim)	Amgen Inc	Accofil®	Accord Healthcare S.L.U.	2014
		Filgrastim Hexal®	Hexal AG	2009
		Grastofil®	Accord Healthcare, SLU	2013
		Nivestim®	Pfizer Europe MA EEIG	2010
		Ratiograstim®	Ratiopharm GmbH	2008
		Tevegrastim®	Teva GmbH	2008
		Zarzio®	Sandoz GmbH	2009
Neulasta® (pegfilgrastim)	Amgen Inc	Pelgraz®	Accord Healthcare S.L.U.	2018
		Fulphila®	Mylan S.A.S	2018
		Udenyca®	ERA Consulting GmbH	2018
		Pelmeg®	Mundipharma Corporation (Ireland) Limited	2018
		Ziextenzo®	Sandoz GmbH	2018
		Grasustek®	Juta Pharma GmbH	2019
		Cegfila®	Mundipharma Corporation (Ireland) Limited	2019
		Nyvepria®	Pfizer Europe MA EEIG	2020
Eprex®/Erypo® (epoetin α)	Janssen‐Cilag GmbH	Abseamed®	Medice Arzneimittel Pütter GmbH Co. KG	2007
		Binocrit®	Sandoz GmbH	2007
		Epoetin‐ α hexal®	Hexal AG	2007
		Retacrit®	Pfizer Europe MA EEIG	2007
		Silapo®	Stada Arzneimittel AG	2007

#### 2.1.3 Monoclonal antibodies

##### Bevacizumab

**FIGURE 5:**
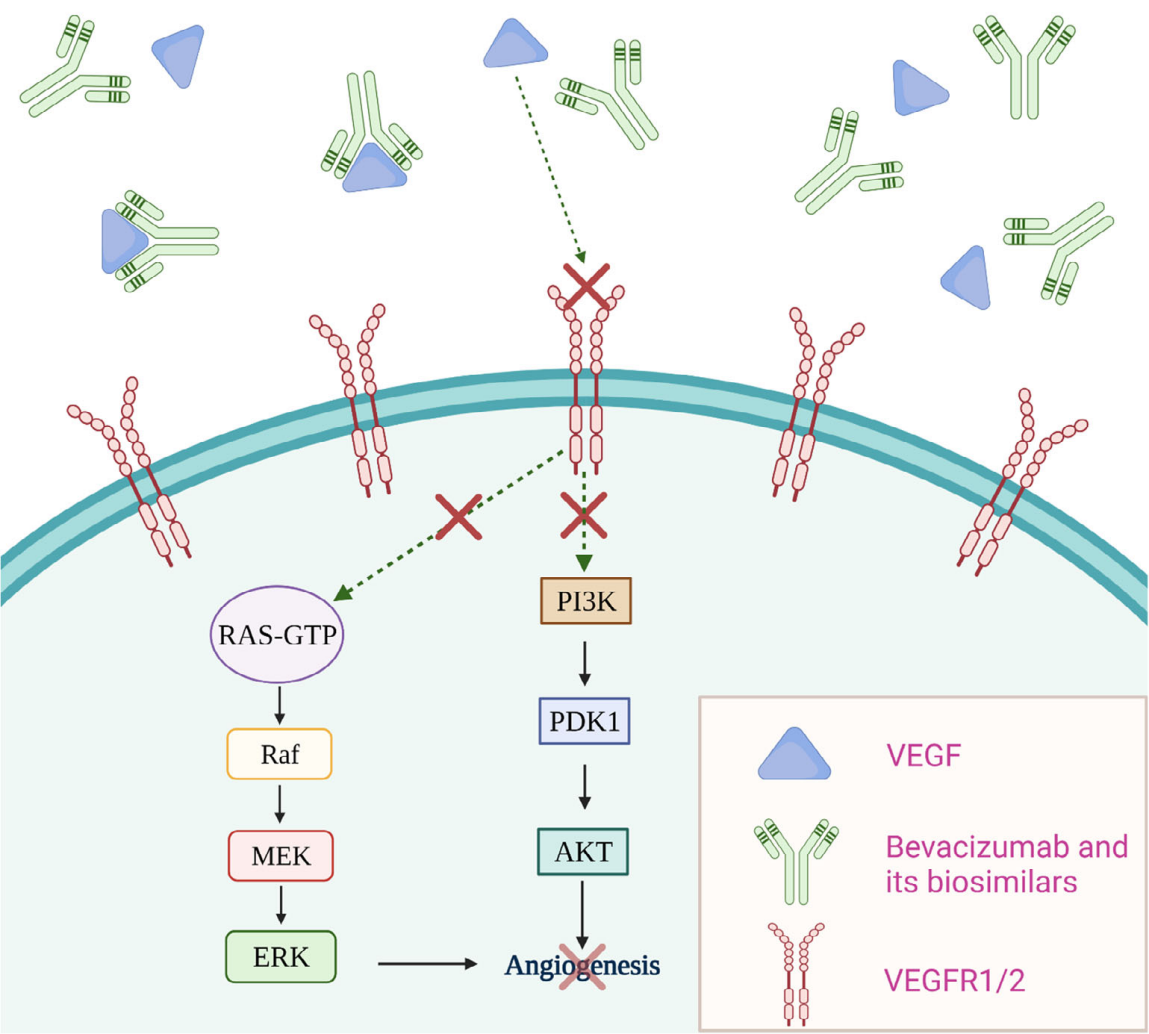
Bevacizumab and its biosimilars' mechanism of action. Upon entry, bevacizumab and its biosimilars bind to VEGF, thereby inhibiting the interaction between VEGF and VEGFR1/2. This in turn blocks angiogenesis signaling pathways.

Bevacizumab is a recombinant humanized monoclonal antibody, that targets vascular endothelial growth factor (VEGF‐A) and inhibits the formation of new blood vessels (angiogenesis) and the growth of new tumors (Figure 5).^89^ Avastin® (Bevacizumab) is used for various indications including metastatic colorectal cancer (mCRC), non‐squamous non–small cell lung cancer (NSCLC), glioblastoma, metastatic renal cell carcinoma (mRCC), and persistent, recurrent, or metastatic carcinoma of the cervix either as a single agent or in combination with chemotherapy/biologic response modifier.^90^ The patent of Avastin® in the United States expired in 2019 whereas in Europe the patent will expire in 2022.^91^ Currently, two biosimilars of Avastin® (bevacizumab) are available in the United States including Mvasi® (bevacizumab‐awwb)^92^ and Zirabev® (bevacizumab‐bvzr).^93^ Both of these biosimilars^94^, ^95^ along with few others including Aybintio®,^96^ Equidacent®,^97^ Oyavas®,^98^ and Alymsys®^99^ are approved in Europe. However, these biosimilars could face a delay in reaching the market until relevant patents and regulatory exclusivities expire.^100^

##### Rituximab

**FIGURE 6:**
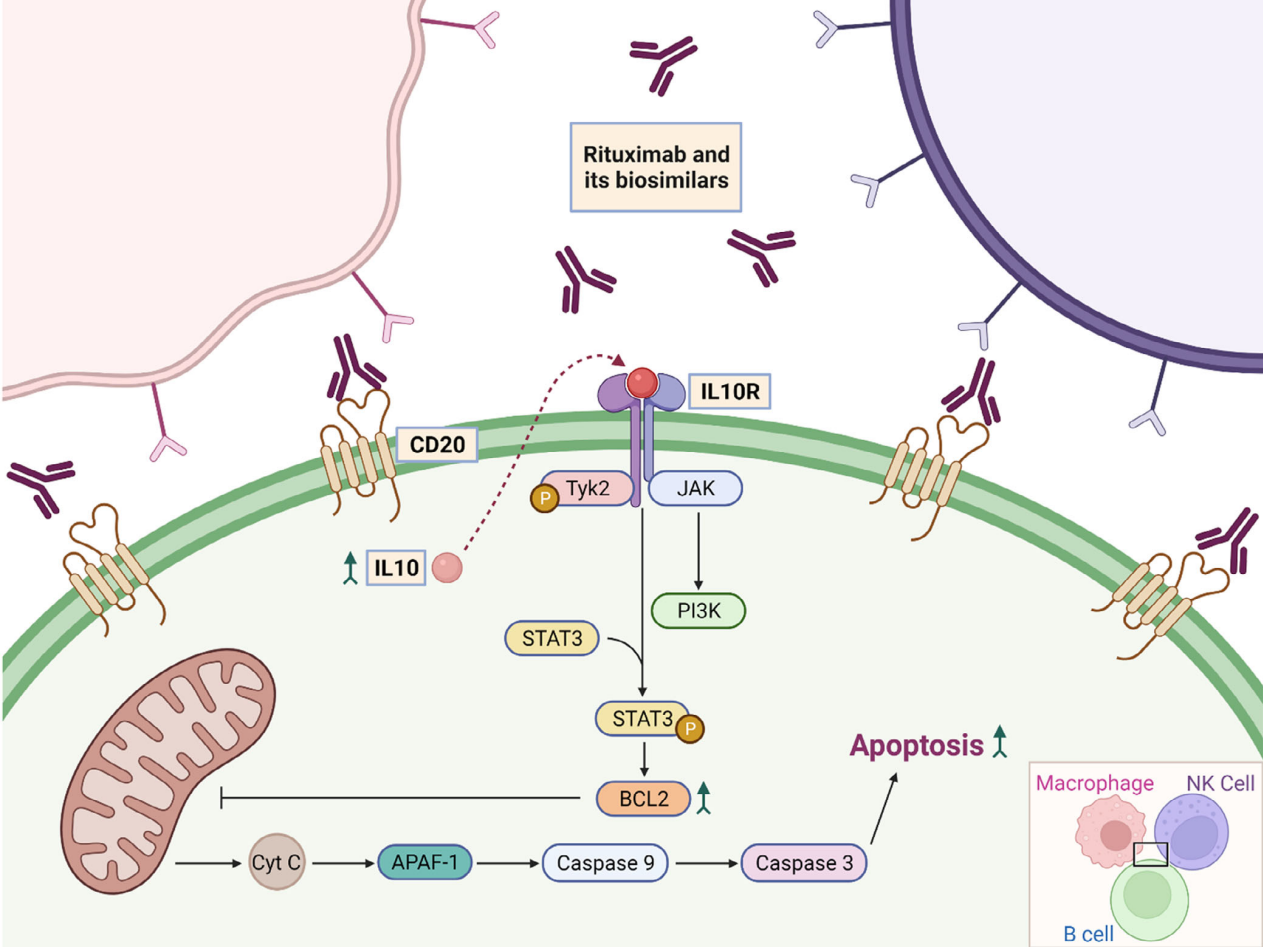
Cell death mechanisms involved in rituximab and its biosimilars' b‐cell binding. This antibody and its biosimilars induce an apoptotic control pathway through binding with CD20 receptors. Cellular binding also facilitates two other cell death pathways including phagocytosis and lysis by natural killer cells.

Rituxan® (Rituximab) is a genetically engineered chimeric human monoclonal antibody that targets the CD20 antigen, found on the surface of B lymphocytes. By binding to the CD20 antigen, rituximab and its biosimilars increase IL‐10 and B‐cell lymphoma‐2 (Bcl‐2) thereby inducing cellular apoptosis^101^ (Figure 6). Rituxan® is indicated for treating patients with Non‐Hodgkin's Lymphoma (NHL) and Chronic Lymphocytic Leukemia (CLL).^102^ It is indicated for use as a single agent for relapsed/refractory, low‐grade/ follicular CD20‐positive, B‐cell NHL, and in patients with non‐progressing, low‐grade, CD20‐positive, B‐cell NHL after first‐line cyclophosphamide, vincristine, and prednisolone (CVP) chemotherapy. It is also used in combination with chemotherapy in patients with previously untreated follicular & diffuse large B‐cell, CD20 positive B‐cell NHL, and as single‐agent maintenance therapy in patients who achieved a complete/partial response to Rituxan®. In patients with CLL, Rituxan® is used in combination with chemotherapeutics: fludarabine and cyclophosphamide (FC).^103^ The patent for Rituxan® (rituximab) in the United States expired in 2016^104^ which led to the development of biosimilars Truxima® (rituximab‐abbs),^105^ Ruxience® (rituximab‐pvvr),^106^ and recently Riabni® (rituximab‐arrx).^107^ Even in Europe, the patent for MabThera® (rituximab) expired in 2013,^104^ and six biosimilars for rituximab were approved by EMA including Blitzima®,^108^ Truxima®,^109^ Ruxience®,^110^ Riximyo®,^111^ Rixathon®^112^ and Ritemvia®.^113^

##### Trastuzumab

**FIGURE 7:**
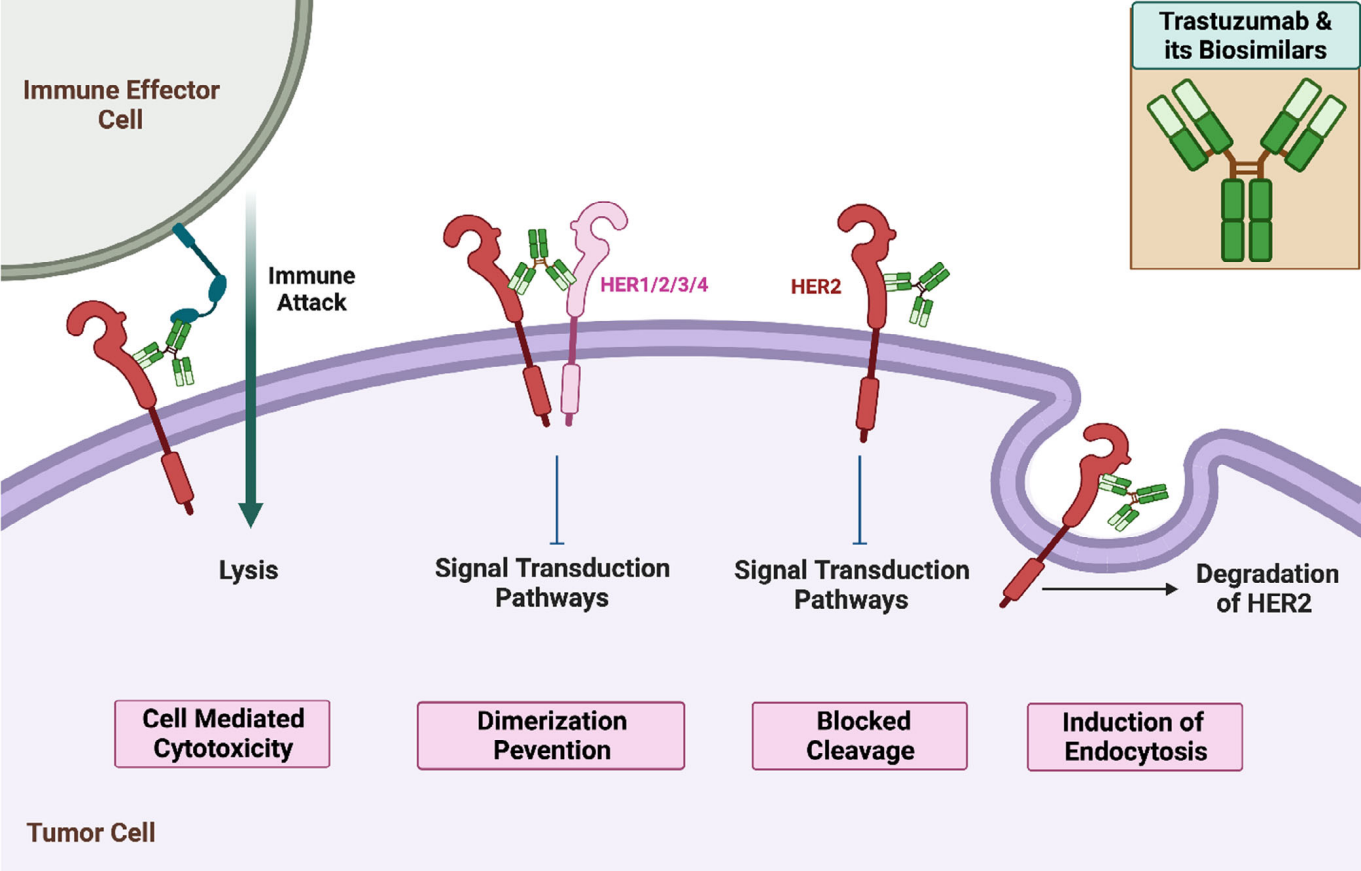
Trastuzumab and its biosimilars' mechanisms of action. (1). Upon binding to HER2 receptors, endocytosis and further degradation of the receptor occurs. This antibody and its biosimilars also (2) prevent receptor cleavage and (3) dimerization with other HER receptors. (4) Cell mediated cytotoxicity is also induced through dual binding with HER2 receptors and immune effector cells.

Herceptin® (Trastuzumab) is a humanized monoclonal antibody that selectively binds to the extracellular domain of the human epidermal growth factor receptor 2 protein (HER2).^114^ The effects of trastuzumab and its biosimilars binding to HER2 receptors are presented in Figure 7. Herceptin® is indicated for patients with (a) metastatic HER2‐overexpressing breast cancer either as a single agent or in combination with paclitaxel (b) metastatic HER2‐overexpressing gastric cancer in combination with cisplatin and capecitabine/5‐fluorouracil and (c) HER2‐overexpressing breast cancer as an adjuvant treatment in combination with chemotherapeutics or as a single agent following multi‐modality anthracycline‐based treatment.^115^ The patent of Herceptin® (Trastuzumab) in the United States expired in 2019 whereas in Europe the patent expired in 2014.^116^ Ogivri® (trastuzumab‐dkst) was the first biosimilar to Herceptin® (Trastuzumab) to be approved by the FDA. Later in United States four more biosimilars including Herzuma® (trastuzumab‐pkrb),^117^ Trazimera® (trastuzumab‐qyyp),^118^ Ontruzant® (trastuzumab‐dttb),^119^ Kanjinti® (trastuzumab‐anns)^120^ were approved. Ontruzant®,^121^ Herzuma®,^122^ Trazimera®^123^ Kanjinti®,^124^ and Ogivri®^125^ are also approved in Europe. Recently, EMA approved another biosimilar for trastuzumab namely Zercepac®.^126^ Tables 3 and 4 lists the biosimilar drugs approved for monoclonal antibodies‐ Bevacizumab, Rituximab & Trastuzumab by FDA and EMA respectively.

**TABLE 3:** FDA approved mAB biosimilars in oncology

Reference Biologic (Active substance)	Reference Biologic manufacturer(s)	Biosimilar (active substance)	Biosimilar manufacturer	Approval date
Avastin® (Bevacizumab)	Genentech, Inc.	Mvasi® (bevacizumab‐awwb)	Amgen Inc	2017
		Zirabev® (bevacizumab‐bvzr)	Pfizer Inc.	2019
Rituxan® (Rituximab)	Genentech, Inc.	Truxima® (rituximab‐abbs)	Celltrion, Inc	2018
		Ruxience® (rituximab‐pvvr)	Pfizer Ireland Pharmaceuticals	2019
		Riabni® (rituximab‐arrx)	Amgen, Inc	2020
Herceptin® (Trastuzumab)	Genentech, Inc	Ontruzant® (trastuzumab‐dttb)	Samsung Bioepis Co., Ltd	2019
		Trazimera® (trastuzumab‐qyyp)	Pfizer Inc	2018
		Herzuma® (trastuzumab‐pkrb)	Celltrion, Inc	2018
		Kanjinti® (trastuzumab‐anns)	Amgen Inc	2019
		Ogivri® (trastuzumab‐dkst)	Mylan GmbH	2017

**TABLE 4:** EMA approved mAB biosimilars in oncology

Reference Biologic (Active substance)	Reference Biologic manufacturer(s)	Biosimilar (active substance)	Biosimilar manufacturer	Approval date
Avastin® (bevacizumab)	Roche Registration GmbH	Mvasi®	Amgen Technology (Ireland) UC	2018
		Zirabev®	Pfizer Europe MA EEIG	2019
		Aybintio®	Samsung Bioepis NL B.V.	2020
		Equidacent®	Centus Biotherapeutics Europe Limited	2020
		Oyavas®	STADA Arzneimittel AG	2021
		Alymsys®	Mabxience Research SL	2021
MabThera® (rituximab)	Roche Registration GmbH	Blitzima®	Celltrion Healthcare Hungary Kft.	2017
		Truxima®	Celltrion Healthcare Hungary Kft.	2017
		Ruxience®	Pfizer Europe MA EEIG	2020
		Riximyo®	Sandoz GmbH	2017
		Rixathon®	Sandoz GmbH	2017
		Ritemvia®	Celltrion Healthcare Hungary Kft.	2017
Herceptin® (Trastuzumab)	Roche Registration GmbH	Ontruzant®	Samsung Bioepis NL B.V.	2017
		Trazimera®	Pfizer Europe MA EEIG	2018
		Herzuma®	Celltrion Healthcare Hungary Kft.	2018
		Kanjinti®	Amgen Europe B.V.	2018
		Ogivri®	Mylan S.A.S	2018
		Zercepac®	Accord Healthcare S.L.U.	2020

#### 2.1.4 Pharmacoeconomics of biosimilars in oncology

**FIGURE 8:**
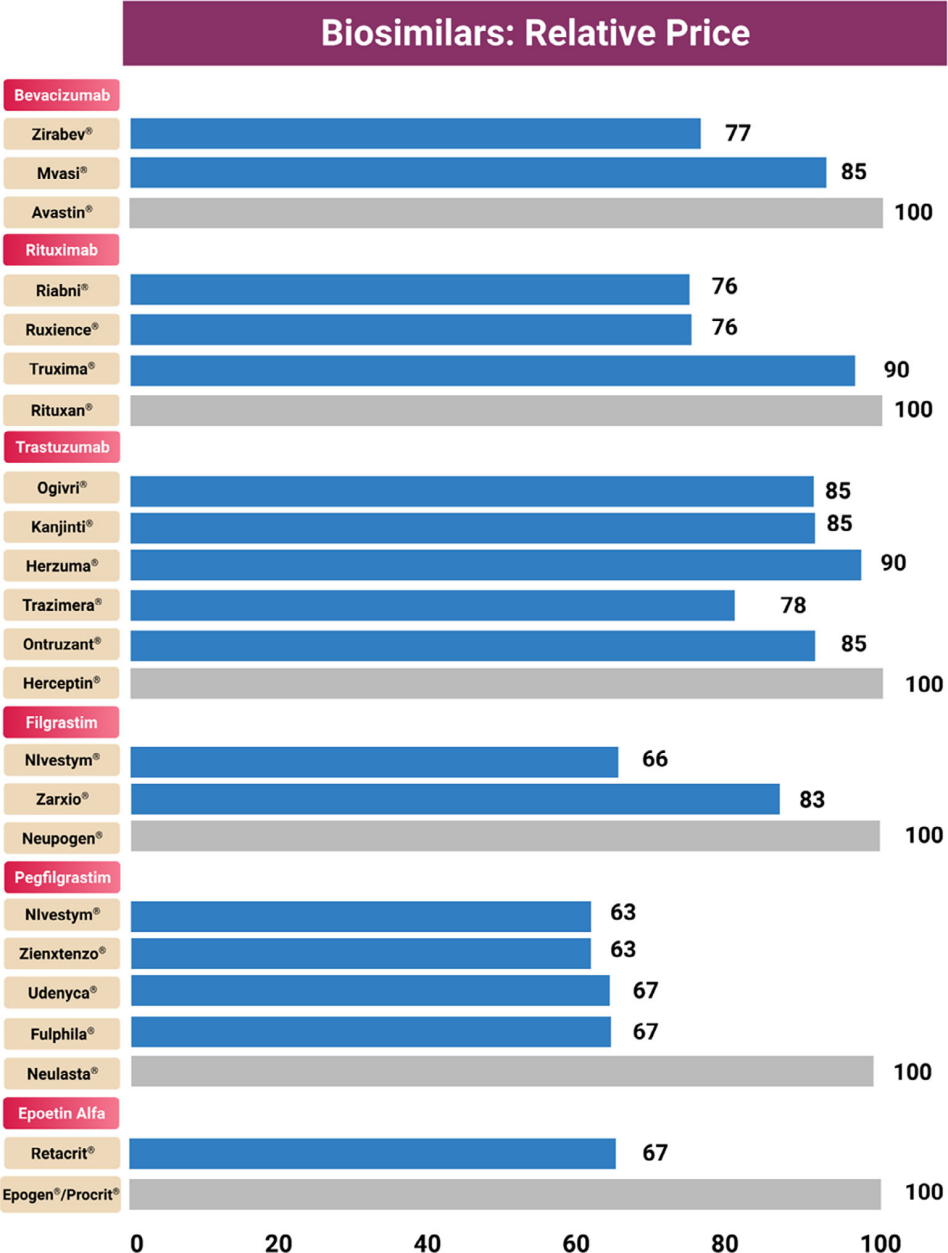
Relative prices of oncology biosimilars and their biosimilars

A comparative cost analysis was performed using the average wholesale price (AWP) per unit of biologics and biosimilars. The prices of these drugs in the United States were accessed from Red Book® through the database, Micromedex. The current interpretation is based on the drug prices in June 2021. A comparison of the AWP costs between biosimilars and their reference products is provided in Table 5 and the relative biosimilar prices are shown in Figure 8.

**TABLE 5:** Biologic and Biosimilar Average wholesale price per unit (AWP) in US$, June 2021

Reference Biologic (Active substance)	Biosimilar	Biosimilar, average wholesale price per unit	Reference product, average wholesale price per unit
Avastin® (Bevacizumab)	Mvasi®	25 mg/ml vial: $203.22	25 mg/ml vial $239.08
	Zirabev®	25 mg/ml vial: $184.02	
Rituxan® (Rituximab)	Truxima®	10 mg/ml vial: $101.46	10 mg/ml vial: $112.74
	Ruxience®	10 mg/ml vial: $86.01	
	Riabni®	10 mg/ml vial: $86.01	
Herceptin® (Trastuzumab)	Ontruzant®	150 mg PDS: $1589.59	150 mg PDS: $1870.10
	Trazimera®	150 mg PDS: $1453.32	
	Herzuma®	150 mg PDS: $1683.00	
	Kanjinti®	150 mg PDS: $1584.54	
	Ogivri®	150 mg PDS: $1589.59	
Neupogen® (Filgrastim)	Zarxio®	480 mcg/0.8 ml vial: $658.47	480 mcg/0.8 ml vial: $797.15
	Nivestym®	480 mcg/0.8 ml vial: $525.60	
Neulasta® (pegfilgrastim)	Fulphila®	6 mg/0.6 ml vial: $8350	6 mg/0.6 ml vial: $12462.11
	Udenyca®	6 mg/0.6 ml vial: $8350	
	Ziextenzo®	6 mg/0.6 ml vial: $7851.06	
	Nyvepria®	6 mg/0.6 ml vial: $7850	
Epogen/Procrit (epoetin α)	Retacrit®	2000 u/ml vial: $26.47	2000 u/ml vial: $39.79

For bevacizumab, the percentage savings with biosimilars ranged from 15% to 23%. Among bevacizumab biosimilars, the savings were significantly higher with Zirabev® when compared to the originator product, Avastin. In the case of rituximab biosimilars, the percentage savings ranged from 10% to 23.7%. Biosimilars including Ruxience® and Riabni® offered greater savings and are currently the most cost‐effective alternatives to Rituxan®. With Herceptin biosimilars, the savings ranged from 15% to 22.2% with the highest cost savings observed with Trazimera®. In addition to these biosimilars, the biosimilars for supportive cancer care agents also provide significant savings when compared to their reference products. The savings range from 17.3% to 34% with filgrastim biosimilars, 33 to 37% with pegfilgrastim biosimilars, and 33.5% with Epogen biosimilar.

## 3 BIOSIMILARS IN CLINICAL TRIALS

**FIGURE 9:**
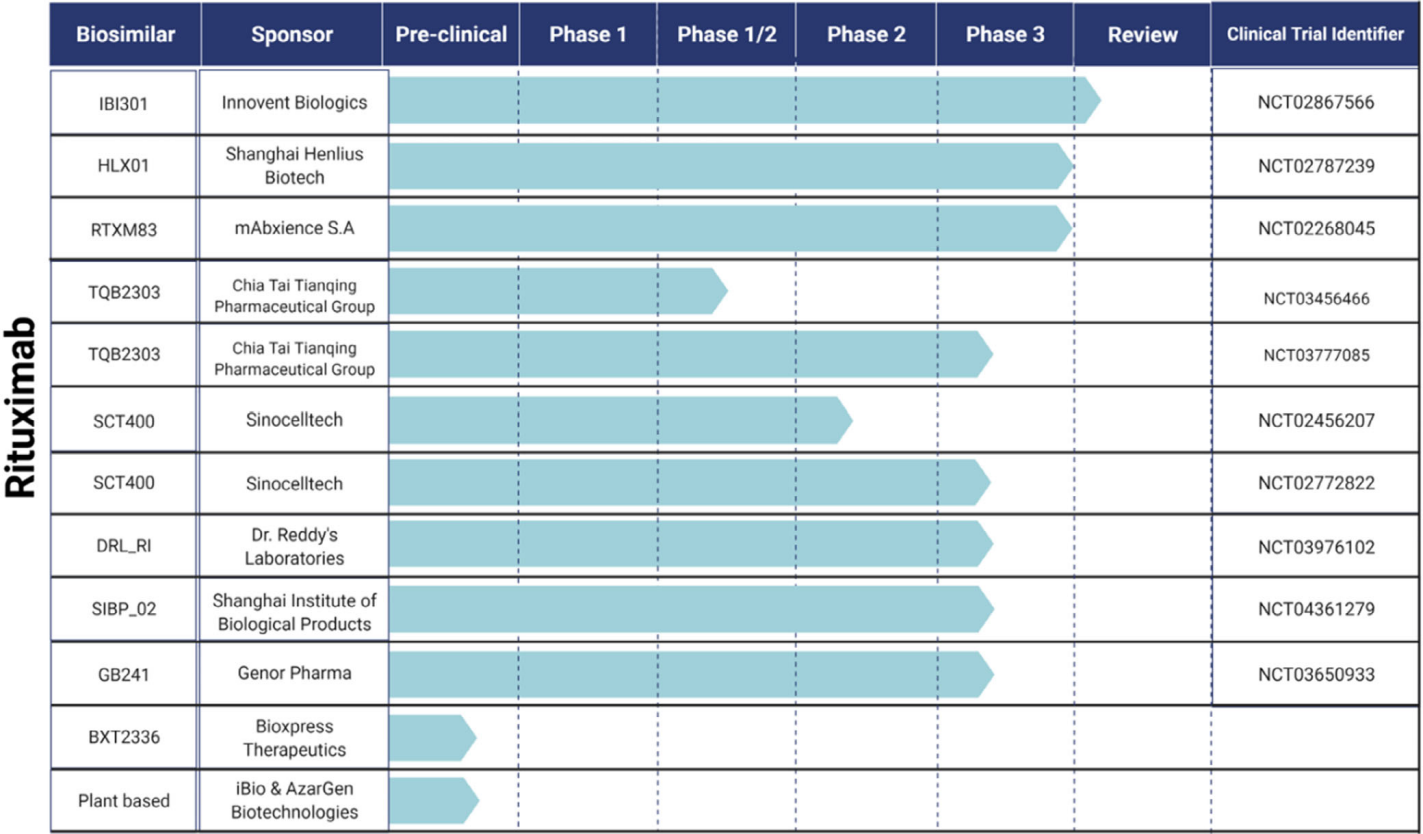
Prospective biosimilar candidates of Rituximab that are in different phases of clinical trials.

**FIGURE 10:**
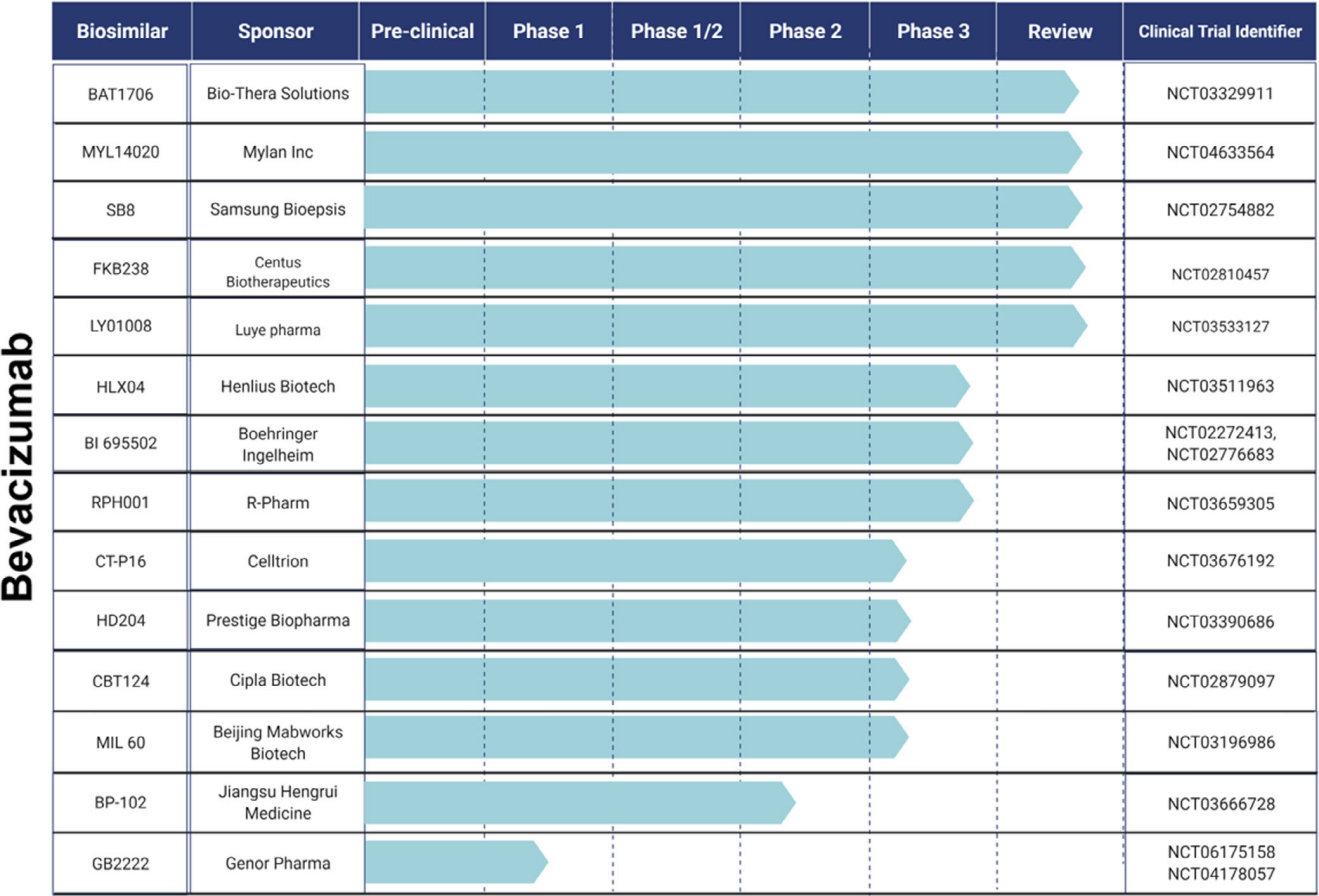
Prospective biosimilar candidates of Bevacizumab that are in different phases of clinical trials.

**FIGURE 11:**
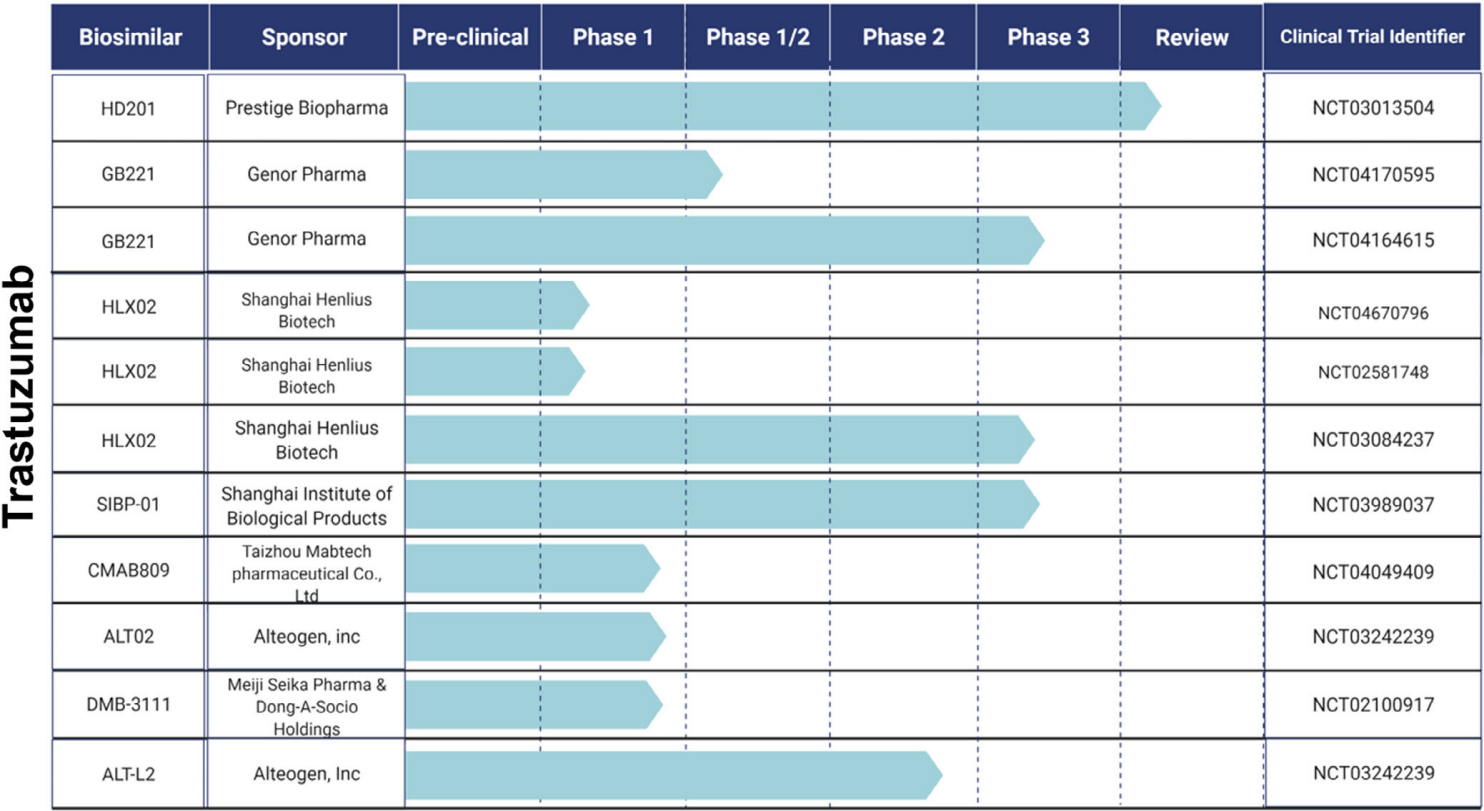
Prospective biosimilar candidates of Trastuzumab that are in different phases of clinical trials.

Several biosimilar candidates are being globally developed, investigated, and are currently in various stages of clinical development & regulatory approval. This section summarizes the studies involving prospective biosimilar candidates in oncology based on their clinical research progress. Figures 9, 10, and 11 provide the clinical trial information of the respective candidates.

-
**Candidates awaiting regulatory agency response** : Few manufacturers have submitted Biologics License Application (BLA) for their proposed bevacizumab biosimilar candidates (BAT1706, MYL‐1402O, SB8, and FKB238) that are currently under FDA review. Bio‐thera (BAT1706) seeks approval for its candidate use in treating non‐small cell lung cancer, recurrent glioblastoma, metastatic renal cell carcinoma, persistent/recurrent/metastatic cervical cancer, and mCRC in combination with chemotherapy.^127^ The application is based on the positive results from preclinical, pharmacokinetic, and international, multicenter phase 3 comparative safety & efficacy studies.^128^ Mylan and Biocon Limited (MYL‐1402O) seeks approval for the same indications as to the originator, bevacizumab. The application is supported by phase 3 findings from comparative safety, efficacy, and immunogenicity evaluating global study^129^ This company also submitted its marketing authorization application to the EMA.^130^, ^131^
  Luye Pharma has announced that the marketing authorization application for its biosimilar candidate (LY01008) has been accepted by the China Center for Drug Evaluation of the National Medical Products Administration (NMPA). The application was based on data generated from two comparative studies: a pharmacokinetics study in healthy volunteers and a safety & efficacy study in metastatic/recurrent non‐squamous non‐small cell lung cancer patients. Both of these studies compared the biosimilar candidate to its reference drug, Avastin® and have met their pre‐defined primary endpoints.^132^ Other biosimilar candidates SB8 (Samsung Bioepsis) and FKB238 (Centus Biotherapeutics) have already received marketed authorization from the EU and are available as Aybintio®^96^ and Equidacent®^97^ respectively. The BLA of these candidates was accepted by the FDA in 2019 and the manufacturers are looking forward to launching their products in the US.^131^, ^133^ HD201 from Prestige biopharma, a biosimilar candidate to trastuzumab has completed phase 3 studies in HER2 positive breast cancer patients. The marketing application of biosimilar HD201 has also been accepted by the EMA.^134^ Innovent Biologics, Inc and Eli Lilly have announced that NMPA has accepted their New Drug Application (NDA) for IBI301, a biosimilar candidate to rituximab. The application is based on the clinical data obtained from studies including Phase 3‐ safety & efficacy of IBI301 along with chemotherapy and phase 1 pharmacokinetics & safety assessment in patients with untreated CD20‐positive diffuse large B‐cell lymphoma (DLBCL).^135^
-
**Candidates that have completed Phase 3 studies** : HLX01 by Shanghai Henlius Biotech, completed a dose‐escalation,^136^ safety, pharmacokinetic & pharmacodynamic studies^137^ in patients with CD20 positive B‐cell lymphomas in comparison to Mabthera®. A phase 3 study evaluating safety and efficacy of the candidate in combination with chemotherapy was also investigated.^138^ Currently, a follow‐up study of HLX01 to determine the overall survival (OS) and progression‐free survival (PFS) is underway.^139^ RTXM83 (mAbxience S.A) is another biosimilar candidate, to Mabthera® that has completed phase 3 trials in patients with DLBCL.^140^ Biosimilar HLX04 from Henlius Biotech has met the primary end point in phase 3 safety, efficacy, and immunogenicity studies in comparison to its reference drug, bevacizumab. This study investigated the candidate in combination with oxaliplatin and fluoropyrimidine‐based chemotherapy (XELOX or mFOLFOX6) as first‐line treatment in patients with mCRC.^141^ Also, HLX04 combined with Henlius's anti‐PD‐1 monoclonal antibody (mAb) HLX10 is being investigated for the treatment of different types of cancer including advanced solid tumors (phase 1)^142^ and advanced hepatocellular carcinoma (HCC) (phase 2).^143^ Another biosimilar candidate of bevacizumab, BI 695502 by Boehringer Ingelheim has completed two comparative phase 3 studies. The candidate was evaluated in combination with chemotherapeutics as a first line treatment and as maintenance therapy in patients with lung cancer & mCRC respectively.^144^, ^145^ Two biosimilar candidates of R‐Pharm, RPH001 (reference drug: bevacizumab) and RPH‐002 (reference drug cetuximab) have also completed phase 3 studies and the results are yet to be updated by the company.^146^
-
**Candidates currently undergoing Phase 3** : TQB2303, is a rituximab biosimilar candidate of Chia Tai Tianqing Pharmaceutical Group Co., Ltd and it is currently being investigated in two clinical studies (Phase 1/2 & Phase 3) involving CD20‐positive DLBCL patients.^147^, ^148^ SCT400 by Sinocelltech Ltd has completed a phase 1 safety and efficacy study in patients with B‐cell Non‐Hodgkin's lymphoma.^149^ The company has initiated phase 2^150^ and phase 3^151^ studies comparing the candidate to the reference drug rituximab. Few other biosimilar candidates to rituximab that are undergoing phase 3 trials to demonstrate their equivalent efficacy include DRL_RI,^152^ by Dr. Reddy's Laboratories Limited, SIBP‐02^153^ by Shanghai Institute of Biological Products, and GB241 by Genor Pharma.^154^ GB221, another candidate of Genor Biopharma completed a safety & pharmacokinetic study following single‐dose administration in patients with metastatic breast cancer. This study is conducted in comparison to the reference drug, Herceptin®.^155^ Phase I/II studies of single/multiple doses of GB221^156^ and a phase 3 study to evaluate progression‐free survival (PFS) using combinational therapy are underway.^157^ HLX02, (Henlius Biotech) is being investigated in three comparative safety & immunogenicity clinical studies including two phase 1 studies in healthy volunteers^158^, ^159^ and a phase 3 study in breast cancer patients.^160^ This trastuzumab candidate has already been approved in the EU (Zercepac®).^126^ The company has also entered into a collaboration with Accord Healthcare, US granting an exclusive right to develop and commercialize in the US and Canada.^161^ Another biosimilar candidate of trastuzumab undergoing comparative phase 3 studies in breast cancer patients include SIBP‐01, which is developed by the Shanghai Institute of Biological Products.^162^ Quite a few prospective bevacizumab biosimilars have completed phase one studies and are currently undergoing comparative safety & efficacy (phase 3) studies. These include CT‐P16 by Celltrion,^163^ HD204 by Prestige biopharma^164^ CBT124 by Cipla BioTech^165^ and MIL 60 by Beijing Mabworks Biotech.^166^ All of these biosimilar candidates are evaluated for their use in treating patients with non‐small cell lung cancer. Two biosimilar candidates for the originator Xgeva® are also undergoing phase 3 studies. These include LY01011 by Luye Pharma^164^ and QL 1206 by Qilu Pharmaceuticals.^167^
-
**Candidates in Phase 1 and 2** : Trastuzumab biosimilar candidates including CMAB809 by Taizhou Mabtech Pharmaceutical Co., Ltd,^168^ ALT02 by Alteogen, Inc,^132^ and DMB‐3111 by Meiji Seika Pharma & Dong‐A‐Socio Holdings have completed phase 1 similarity studies.^169^ Another trastuzumab biosimilar candidate of Alteogen Inc, ALT‐L2^170^ has completed global phase 2 testing and is getting ready for phase 3 studies.^171^ BP‐102 by Jiangsu‐Hengrui‐Medicine is currently in phase 2 clinical trials. The proposed bevacizumab biosimilar candidate is evaluated in chemotherapy‐naive patients with non‐squamous NSCLC.^172^ Another biosimilar candidate of bevacizumab, GB222 (Genor Pharma) is currently undergoing various phase 1 trials for the treatment of glioblastoma multiforme, non‐squamous non‐small cell lung cancer, and mCRC.^173^ Biosimilar candidate for originator drug denosumab, TK006 is developed by Jiangsu T‐Mab Biopharma Co., Ltd. TK006 is currently being evaluated (phase 1) for its safety upon single, multiple doses in patients with breast cancer‐related bone metastases.^174^, ^175^ Few biosimilar candidates for supportive care agent Neulasta® have completed phase 1 studies. These include INTP5 by Intas Pharmaceuticals, Ltd.^176^ PF‐06881894 by Pfizer,^177^ and B12019 by Cinfa Biotech.^178^ QL0605 by Qilu Pharmaceutical Co., Ltd is another biosimilar for Neulasta®, which is currently undergoing phase 1 studies.^179^
-
**Candidates in preclinical development** : Biosimilar candidates for rituximab that are in the early stages of development include BXT 2336 by Bioxpress therapeutics^180^ & a plant‐based product by iBio & AzarGen Biotechnologies.^181^ Few biosimilar candidates from Prestige biopharma are also in early development. These include PBP1602 (reference drug: aflibercept), PBP1701 (reference drug: ipilimumab), and PBP1801 (reference drug: pertuzumab).^182^ CMAB810 (Taizhou Mabtech Pharmaceutical Co., Ltd) is another biosimilar candidate for reference drug pertuzumab which is in preclinical studies.^183^ Biosimilars for originators Opdivo® and Keytruda® are in active development by NeuClone Ltd.^184^ Furthermore, biosimilar candidates Xdivane, (reference drug Opdivo®) and Spherotide, (reference drug, Decapeptyl®) by Xbrane Biopharma are also in their preclinical studies.^185^

However, few companies have suspended clinical development of their rituximab biosimilar candidates including BI 695500 (Boehringer Ingelheim Pharmaceuticals),^186^ Kikuzubam (Probiomed),^187^ SAIT101 (Archigen Biotech Limited),^188^ TL011 (Teva Pharmaceutical Industries),^189^ JHL1101 (JHL Biotech, Inc)^190^, ^191^ and GP2013 (Sandoz)^192^ either due to the changes in regulatory requirements or marketing decisions.^193^ JHL Biotech, Inc also suspended clinical development of its two other biosimilars candidates including JHL1188 (reference drug: trastuzumab) and JHL1149 (reference drug: bevacizumab) due to legal issues.^190^ Shanghai Henlius Biotech withdrew its rituximab biosimilar candidate HLX01 from phase 3 studies due to strategic reasons. The study was intended to evaluate the candidate safety and efficacy in patients with low tumor burden follicular lymphoma.^194^ For the same reasons, Jiangsu‐Hengrui‐Medicine also withdrew its bevacizumab candidate BP‐102, from a phase 2 evaluation in patients with mCRC.^195^

## 4 CONCLUSIONS AND FUTURE PERSPECTIVES

The use of biosimilars is rapidly evolving and will continue to play an important role in the future care of cancer patients.^196^ Many biosimilars are expected to be available in the coming years and their use will largely depend on patient and provider acceptance, which is in turn based on an adequate understanding of the safety and efficacy of these agents in cancer treatment.^23^ Therefore, education of patients and providers on various aspects of biosimilars is necessary to increase confidence in biosimilars and for their successful incorporation in oncology practice. Furthermore, rigorous regulatory frameworks and close post‐marketing monitoring of these drugs are required to ensure their safety and efficacy in a real‐world setting.^64^

## AUTHOR CONTRIBUTIONS

**Rinda Devi Bachu:** Conceptualization (equal); data curation (equal); methodology (equal); resources (equal); visualization (equal); writing – original draft (equal). **Mariam Abou‐Dahech:** Formal analysis (equal); methodology (equal); software (equal); validation (equal); visualization (equal). **Swapnaa Balaji:** Data curation (supporting); formal analysis (supporting); investigation (supporting); methodology (supporting); validation (supporting). **Sai H.S. Boddu:** Formal analysis (supporting); methodology (supporting); visualization (supporting); writing – review and editing (supporting). **Samson Amos:** Validation (supporting); writing – review and editing (supporting). **Vishal Singh:** Data curation (supporting); methodology (supporting); visualization (supporting). **R. Jayachandra Babu:** Conceptualization (supporting); data curation (supporting); project administration (supporting); supervision (supporting); visualization (equal); writing – review and editing (equal). **Amit K. Tiwari:** Conceptualization (equal); formal analysis (equal); funding acquisition (equal); investigation (equal); project administration (equal); resources (equal); software (equal); supervision (equal); validation (equal); visualization (equal); writing – original draft (equal); writing – review and editing (equal).

## FUNDING INFORMATION

The manuscript has been supported in part by Susan G. Komen Breast Cancer Foundation (CCR18548498 to Amit K. Tiwari) and Department of Defense (W81XWH210053 to Amit K. Tiwari). The views expressed in this article are those of authors and may not reflect the official policy or position of the Department of the Army, Department of Defense or the US Government or Susan G. Komen Breast Cancer Foundation.

## CONFLICT OF INTEREST

The authors declare that the research was conducted in the absence of any commercial or financial relationships that could be construed as a potential conflict of interest.

## ETHICS STATEMENT

This review did not require any ethical clearance. However, all participants practiced highest ethical standards as mandated by National Institute of Health throughout the preparation of this manuscript.

## Data Availability

Not applicable.
